# Supplementary figures and images for: A pseudomolecule assembly of the Rocky Mountain elk genome
Source: PLoS One. 2021 Apr 28;16(4):e0249899. doi: 10.1371/journal.pone.0249899 (PMC8081196; doi:10.1371/journal.pone.0249899)

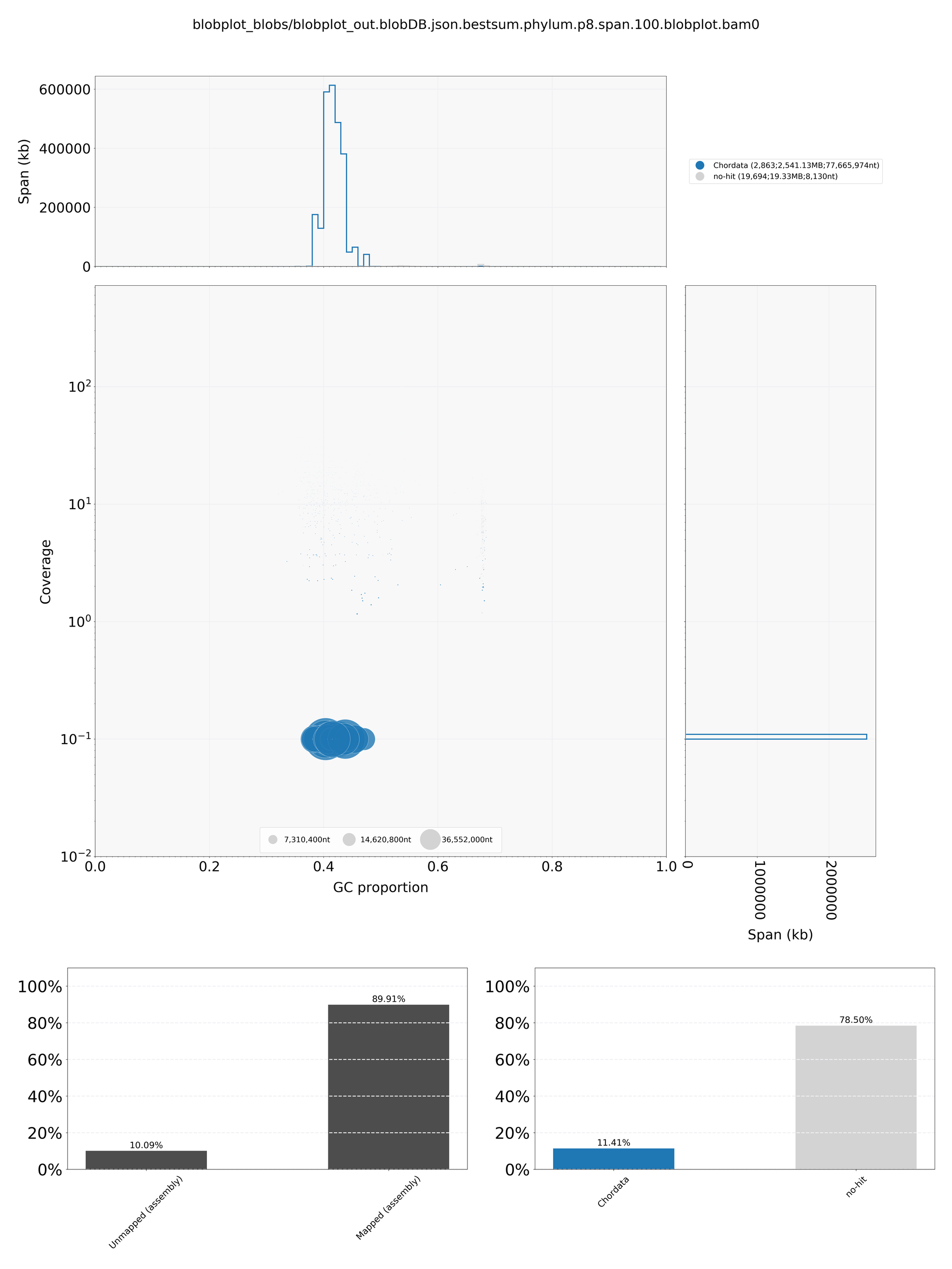

Supplement: S1 Fig — (TIF) [file pone.0249899.s007.tif]
