# Supplementary material for: A pseudomolecule assembly of the Rocky Mountain elk genome
Source: PLoS One. 2021 Apr 28;16(4):e0249899. doi: 10.1371/journal.pone.0249899 (PMC8081196; doi:10.1371/journal.pone.0249899)
Supplement: S1 File — (DOCX) [file pone.0249899.s008.docx]

# Mitochondrial Genome

The mitochondrial genome is 16,429 bases long with 39 gene models, including tRNAs. Three potentially anomalous and nearly complete mitochondrial integrations were identified in the genome ranging from 93-99% identical and nearly full-sized on Chromosome_02, Chromosome_12, and Chromosome_23.

# Blobtools Contamination Check

Supplemental Figure 1.


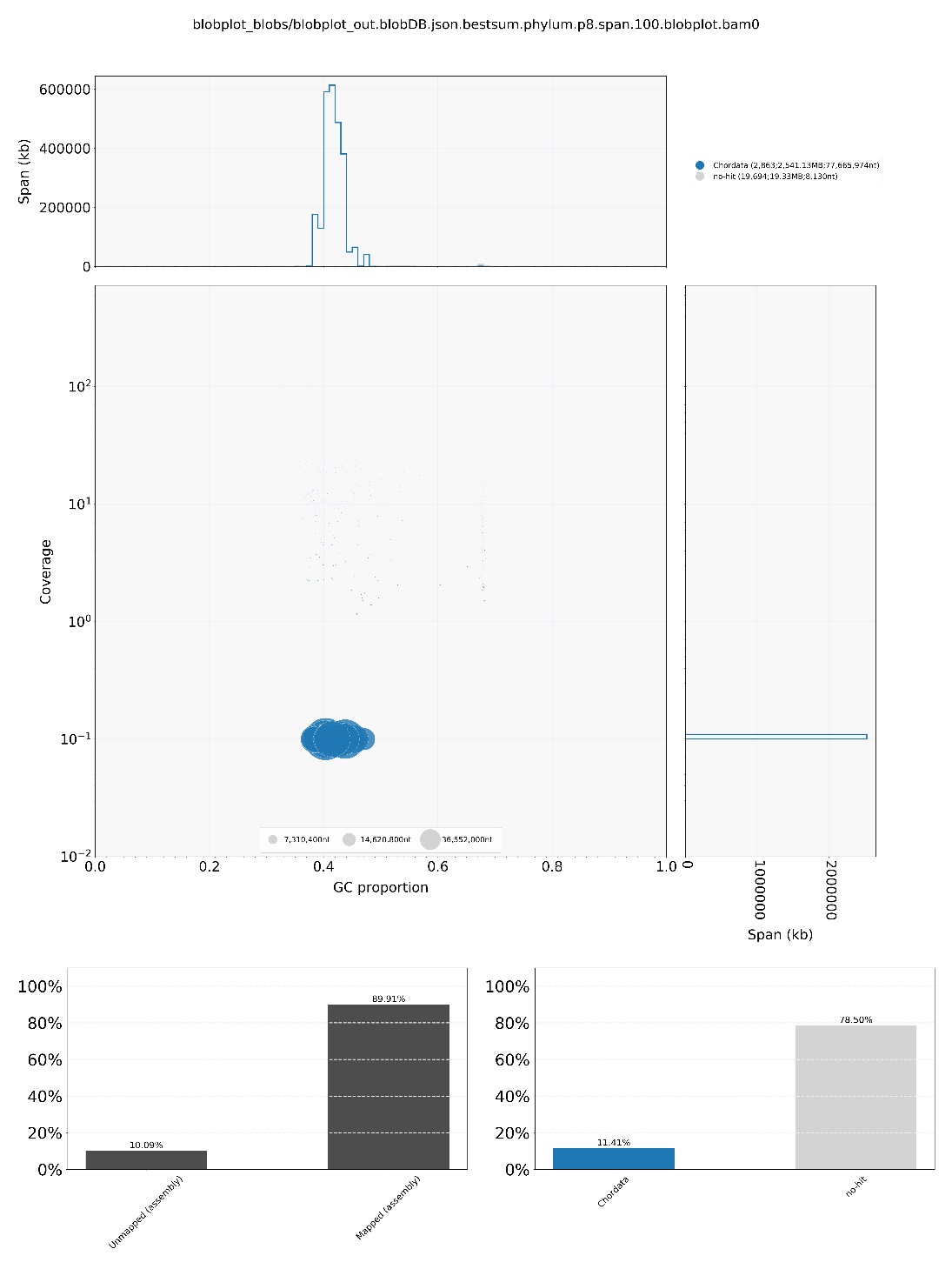


Blobtools analysis of C. canadensis genome using PacBio subreads for mapping and NCBI NT database.
