# Supplementary material for: A pseudomolecule assembly of the Rocky Mountain elk genome
Source: PLoS One. 2021 Apr 28;16(4):e0249899. doi: 10.1371/journal.pone.0249899 (PMC8081196; doi:10.1371/journal.pone.0249899)
Supplement: Attachment — Submitted filename: ResponseToReviewers2.docx [file pone.0249899.s010.docx]

Dear Editor,

We thank the reviewers for their time and effort in reviewing this manuscript, as well as improving the clarity of our analyses. Please see directed comments below.

Sincerely,

Rick Masonbrink

I believe section describing the putative gene loss could still be further improved. For example, how does the elk genome sequence look on the regions these genes were expected to locate? Are there gaps? Are these contiguous regions? It would also be very interesting to see if there are still remnants of these genes in the elk genome? Or are these genes located at the boundaries of chromosomal inversions, for example? Nonetheless, I do understand that the last two questions might require more time to investigate. The information about the sequence contiguity around these putative lost genes, however, would address the concerns of misassembly in these regions.

We have refurbished the gene loss analysis to have more clarity and direct interpretations from the data. The new analysis includes the sequence information of these gene loss regions, and the corresponding region for cattle (Table s6). Initially we investigated these gene losses with just tBLASTn, but have since investigated these regions extensively with BLASTn, adding significant depth to the information on these regions. Please see the methods at line 189-196 to see that tBLASTn, blastn of cattle sequences that hit but were missing in elk, and blastn of cattle sequence hits + 20bp on each side BLastn to elk, and then extracted those sequences in cattle with +100bp borders surrounding for mapping RNAseq and Pacbio reads. These analyses definitely added depth to where these genes may have gone, how they were modified, and/or lost. However, we did not find the remnants or borders of these genes at the borders of chromosomal fissions/fusions or with a verified chromosomal rearrangements. We hope that this additional analysis will be sufficient to allay reviewer concerns about misassembly and/or an incomplete analysis.
